# Effects of Nasal and Paranasal Sinus Variations on Chronic Otitis Media Development in Pediatric Patients

**DOI:** 10.5152/eurasianjmed.2021.20167

**Published:** 2021-10

**Authors:** Nihal Efe Atila, Kübra Topal, Yunus Emre Bulut, Zülküf Kaya, Berrin Arslan

**Affiliations:** 1 Department of Otorhinolaryngology, Sağlık Bilimleri University Training and Research Hospital, Erzurum, Turkey; 2 Department of Otorhinolaryngology, Kastamonu University School of Medicine, Kastamonu, Turkey; 3 Department Of Public Health, Ankara Health Directorate, Ankara, Turkey; 4 Department of Otorhinolaryngology, Atatürk University School of Medicine, Erzurum, Turkey; 5 Palandöken State Hospital, Erzurum, Turkey

**Keywords:** Chronic otitis media, Eustache tube disfunction, Sinonasal abnormalities, Computer tomography, Children

## Abstract

**Objective:**

Chronic otitis media is an inflammatory disease of the middle ear. The airflow in the nasal passage affects the development of mastoid air cells through the eustachian tube. Nasal and paranasal pathologies and their anatomical variations cause chronic sinonasal inflammation and affect the middle ear mucosa. This study aims to reveal whether the nasal and paranasal sinus variations in pediatric patients are a factor in developing chronic otitis media.

**Materials and Methods:**

Eighty patients, with unilateral chronic otitis media, who were admitted to the otorhinolaryngology clinic between May 2015 and July 2019, were included in this retrospective study. The control group (Group 2) consisted of a total of 51 patients. None of the patients in Group 2 had otorrhea history and no signs of chronic otitis in their otoscopic examination, middle ear pathologies. The patient’s anatomical variations of the nasal cavity and sinuses were evaluated with CT by a radiologist

**Results:**

Nasal septum deviation was found to be 53% in children with chronic otitis media. It was found 31.4% in the control group. Since the *P* value was found to be .04, this rate was considered as significant. Inferior concha hypertrophy was found to be 17.6% in the control group and 38.8% in the group with chronic otitis media, and this rate was considered significant since the *P* value was .035

**Conclusion:**

We detected that septum deviation and inferior concha hypertrophy increased chronic otitis media formation in children. All these studies show that cases causing nasal obstruction, such as septum deviation, have a negative effect on middle ear pressure and increase the rate of ear diseases

## Introduction

Chronic otitis media is an inflammatory disease of the middle ear. In the long term, it causes changes such as perforation, adhesion, and retraction in the tympanic membrane. Genetic factors, environmental factors, anatomical and functional disorders of the eustachian tube, and nasal and paranasal sinus pathologies are predisposing factors.^1^,^2^ The middle ear cavity, the paranasal sinuses, and the eustachian tube are lined with pseudostratified columnar epithelium. There are goblet cells secreting mucus in the middle ear cavity and paranasal sinuses. Pneumotization and drainage of the middle ear are provided by the eustachian tube. Anatomical, physiological obstructions of the nose, and paranasal sinuses affect the function of the middle ear.^3^,^4^ Pathological changes in the nose and paranasal sinuses’ development process can facilitate otitis media formation by affecting the development of mastoid air cells.^3,4^ Anatomical variations in the nasal cavity and paranasal sinuses obstruct the osteomeatal unit and cause nasal congestion, impaired drainage. Haller cell, ager nasi cells, paradoxical middle concha, concha bullosa, septum deviation, and inferior concha hypertrophies can obstruct the nasal passage.^5^ The relationship between middle ear effusion and eustachian tube dysfunction has been revealed, and studies have proven that eustachian dysfunction is the major risk factor for benign middle ear diseases.^3^ Nasal and paranasal pathologies and their anatomical variations causing chronic sinonasal inflammation also affect the middle ear mucosa.^6^ The aeration of mastoid cells begins at the 33rd week of pregnancy proceeds until the age of 8-9.^7^ The airflow in the nasal passage affects the development of mastoid air cells through the eustachian tube.^8^ Anatomical changes such as nasal septum deviation that disrupt nasal airflow, disrupt the nasopharynx, Eustachian tube pressure, and therefore the aeration of mastoid cells, and loss of aeration in mastoid cells constitutes a risk factor for middle ear diseases.This study aims to reveal whether the nasal and paranasal sinus variations in pediatric patients are a factor in developing chronic otitis media.^9^

## Materials and Methods

We retrospectively determined 80 patients with unilateral suppurative and non-suppurative chronic otitis media who were admitted to the Otorhinolaryngology clinic between May 2015 and July 2019. Temporal bone tomography images and demographic and clinical features of the patients were retrieved from Palandöken State Hospital archive. None of the patients had nasal complaints. Patients with a history of nasal surgery, sinonasal tumor, sinonasal polyposis, hemotimpanium, traumatic tympanic membrane perforation, immunodeficiency, cystic fibrosis and patients over 16 years of age were excluded.

Of the 80 patients, 43 were male and 37 were female, and their ages ranged from 5 to 16. The diagnoses of the patients were done according to their otoscopic examinations, audiometry, and CT images.

The control group (Group 2) consisted of a total of 51 patients, 26 males and 25 females with head and neck masses, vascular, lymphatic anomalies, and chronic tonsillitis. None of the patients in Group 2 had an otorrhea history and no signs of chronic otitis in their otoscopic examination, middle ear pathologies.

Tomography images were examined in a routine, standard way. Using PACS (Picture Archiving and Communication Systems), it was ensured that small details were not overlooked. Anatomical variations of the nasal cavity and sinuses were evaluated with temporal CT scans. No additional paranasal CT were taken. Nasal septum deviation, ager nasi cell, onodi cell, middle concha variations, lower concha hypertrophy, frontal sinus aplasia, and superior concha pneumotization were detected. The parameters searched were evaluated as present or absent. The findings were reported by a single radiologist and analyzed retrospectively. The data obtained from the study and control groups were compared statistically. SPSS 18.0 statistical package program (SPSS Inc.; Chicago, IL, USA) was used in the analysis of the data. For the descriptive data in statistical analysis, Kolmogorov–Smirnov was used for the number, rate, mean, ±standard deviation, compatibility of the groups to a normal distribution, Kruskal–Wallis was used for comparing the numerical data between the groups, and chi-square test was used for comparing the data between the groups obtained by counting. The statistical significance level was accepted as *P* < .05. Approval was obtained from the ethics committee of Erzurum Regional Education and Research Hospital for the study, numbered KAEK 2019/09-85.

## Results

The study was performed with 80 patients diagnosed with unilateral chronic otitis media and applied to Otorhinolaryngology polyclinic between May 2015 and July 2019. Hospital records of patients were retrospectively analyzed in terms of temporal bone CTs, paranasal sinus CTs, demographic and clinical features. It consisted of 37 female and 43 male patients. The age average was 10.66.The control group consisted of 26 female and 24 male patients. The age average was determined as 10.45.

Nasal septum deviation was 53% in children with chronic otitis media. It was found 31.4% in the control group. Since *P* value was .04, this rate was considered as significant. Inferior concha hypertrophy was 17.6% in the control group and 38.8% in the group with chronic otitis media, and this rate was considered as significant since *P* value was .035. There was no significant difference in *P* value (greater than .05) in terms of frontal sinus aplasia, onodi cell, secondary middle concha, concha bullosa, and uncinat process pneumotization.

## Discussion

Mastoid bone cells act as air reservoirs in the middle ear. Primarily, they provide aeration of the middle ear,^4-^^10^ and reduction of the volume difference was created by opening the eustachian tube. Mastoid air cells are associated with paranasal sinuses and have similar anatomical, physiological features. The middle ear and sinonasal region are lined with pseudostratified columnar epithelium and have a similar drainage system. Therefore, they can play a role in the development of otitis media of sinonasal pathologies. Because nasal pathologies such as septal deviation and maxillary sinus pathologies decrease the nasal airflow and concomitantly the aeration of the mastoid cells will decrease, it causes otitis media and other autological problems.^10,11^ In the study of Sign et al., they showed a 31% nasal septum deviation in patients with chronic serous otitis media between the ages of 6 and 65.^12^ In the study of Yedeker et al. performed with patients between 11 and 60 years old, they found this rate as 80%.^1^

In a study by Gapalakrishman and Kumar conducted in patients with chronic otitis media aged 18-49 years, the nasal septum deviation rate was found to be 73%.^13^ In the study of Damar et al., they found that the rate of NSD in patients with COM was 40.7%, whereas it was 28.7% in the healthy control group.These results show that ipsilateral nasal pathologies cause eustachian dysfunction, disrupting the ventilation of mastoid cells, and facilitating the formation of chronic otitis media.^14^

In a study conducted by Fijita et al. on 83 adolescent patients with otitis media with effusion, they detected paranasal sinusitis in 49% of patients. They revealed that sinusitis plays a role in the development of otitis media.^15^ According to the study of Tas et al., the development of mastoid air cells is associated with secretory otitis media, acute otitis, and tubal obstructions seen in childhood. The same study showed that severe septum deviation is causing congestion in the nasal cavity of the same side and thus a decrease in the volume of mastoid air cells.^16^ When a tympanoplasty operation is planned for the patient, the middle ear mucosa, eustachian tube functions, and nasopharynx should be evaluated together. Nasal congestions reduce mastoid cell aeration and tympanoplasty success rate by causing eustachian tube dysfunction.^17^ In their study, Maiere and Krebs indicate that septum deviation and treatment of concha hypertrophies before tympanoplasty increased the operation’s success. Kohl et al. also demonstrated the necessity of providing nasal ventilation before tympanoplasty in their study.^18^

All these studies show that pathologies causing nasal obstruction, such as septum deviation, have a negative effect on middle ear pressure and increase the rate of otologic diseases.^19^

In our study, we have found that septum deviation and inferior concha hypertrophy increased the development of chronic otitis media in children.

In conclusion, septal deviation and inferior concha hypertrophy cause nasal obstruction and decrease nasal airflow, resulting in eustachian dysfunction. This facilitates the recurrence of acute otitis media, which lead to the development of chronic otitis media. Proper and prompt management of nasal and paranasal pathologies can decrease chronic otitis media development and improve the treatment and outcomes in cases with chronic otitis media.

**Main Points**

- Nasal pathologies such as septum deviation can cause nasal obstruction.
- Nasal and paranasal pathologies facilitate the development of chronic otitis media.
- Correction of sinonasal pathologies is important in the treatment of chronic ototis media.

## Figures and Tables

**Table 1.** Gender and Age of the Groups

		Control Group (n = 51)		Chronic Otitis Media Group		
		n	%	n	%	*P*
Gender	Female	25	49.0	37	46.3	.95
	Male	26	51.0	43	53.7	
Mean age		Ort	±SD	Ort	±SD	.85
		10.45	**±**3.26	10.66	3.19	

**Table 2.** Nasal and Paranasal Variations

		Control Group (n = 51)		Chronic Otitis Media (n = 80)		
*Variation*		n	%	n	%	*P*
Frontal *sinus* aplasia	**+**	2	3.9	1	1.3	0.60
	**–**	49	96.1	79	98.7	
Onodi cell	**+**	6	11.8	5	6.3	0.42
	**–**	45	88.2	75	93.8	
Secondary middle concha	**+**	2	3.9	1	1.3	0.60
	**–**	49	96.1	79	98.7	
Concha bullosa	**+**	13	25.5	15	18.8	0.44
	**–**	38	74.5	65	81.2	
Haller cell	**+**	10	19.6	10	12.5	0.54
	**–**	41	80.4	70	87.5	
Uncinate process pneumatization	**+**	3	5.9	3	3.8	0.47
	**–**	48	94.1	77	96.2	
Aghernazi cell	**+**	6	11.8	6	7.5	0.59
	**–**	45	88.2	74	92.5	
Middle concha hypertrophy	**+**	4	7.8	4	5.0	0.64
	**–**	47	92.2	76	95.0	
Inferior concha hypertrophy	**+**	9	17.6	31	38.8	**0.035**
	**–**	42	82.4	49	61.2	
No. septal deviation	**+**	16	31.4	43	53.8	**0.041**
	**–**	35	68.6	37	46.3	
Upper concha hypertrophy	**+**	2	3.9	3	3.8	0.54
	**–**	49	96.1	77	96.2	
